# Childhood Maltreatment in Patients Undergoing Bariatric Surgery: Implications for Weight Loss, Depression and Eating Behavior

**DOI:** 10.3390/nu15092046

**Published:** 2023-04-24

**Authors:** Tair Ben-Porat, Simon L. Bacon, Robbie Woods, Annabelle Fortin, Kim L. Lavoie

**Affiliations:** 1 Montreal Behavioural Medicine Centre (MBMC), Centre Intégré Universitaire de Santé et de Services Sociaux du Nord-de-l’Île-de-Montréal (CIUSSS-NIM), Montreal, QC H4J 1C5, Canada; 2 Department of Health, Kinesiology, and Applied Physiology, Concordia University, Montreal, QC H4B 1R6, Canada; 3 Department of Psychology, Concordia University, Montreal, QC H4B 1R6, Canada; 4 Department of Psychology, Université du Québec à Montréal, Montreal, QC H2X 3P2, Canada

**Keywords:** obesity, bariatric surgery, childhood maltreatment, depressive symptoms, eating behavior

## Abstract

We aimed to explore the relationships between childhood maltreatment and changes in weight, depressive symptoms and eating behavior post-bariatric surgery (BS). Participants (*n* = 111, 85% females) were evaluated pre-surgery, and at 6 months (6 M) and 12 months (12 M) post-BS. History of maltreatment was assessed at baseline (Childhood Trauma Questionnaire), and depressive symptoms (Beck Depression Inventory-II) and eating behavior (Dutch Eating Behavior Questionnaire) were assessed at all time points. Participants’ mean age and median BMI were 45.1 ± 11.7 years and 46.7 (IQR 42.4–51.9) kg/m^2^, respectively. Histories of emotional (EA), physical (PA) and sexual abuse (SA) and emotional (EN) and physical (PN) neglect were reported by 47.7%, 25.2%, 39.6%, 51.4% and 40.5%, respectively, with 78.4% reporting at least one form of maltreatment. Changes in weight and depressive symptoms were not different between patients with vs. without a history of maltreatment. However, those with vs. without SA demonstrated limited changes in emotional eating (EE) at 12 M, while those without showed improvements. Conversely, patients with vs. without EN showed greater improvements in external eating (ExE) at 6 M, but differences were no longer observed by 12 M. Results indicate that histories of SA and EN are associated with changes in eating behaviors post-BS and have implications for assessment, monitoring and potential intervention development.

## 1. Introduction

Bariatric surgery (BS) is currently the most effective long-term treatment for severe obesity and its related co-morbidities [1]. However, over the long term, some patients experience negative health and psychosocial outcomes and are not able to maintain their weight loss [2,3]. One of the psychosocial factors that may impact post-surgical outcomes is a history of exposure to interpersonal trauma in the form of violence, abuse or neglect [4]. According to the World Health Organization, childhood maltreatment is defined as “all forms of physical and/or emotional maltreatment, sexual abuse, neglect or negligent treatment, or commercial or other exploitation, resulting in actual or potential harm to the child’s health, survival, development, or dignity in the context of a relationship of responsibility, trust or power” [5]. Maltreatment includes sexual abuse, physical abuse, emotional abuse, neglect and exposure to intimate partner violence [6]. There are variations in the definitions of the different categories of childhood maltreatment, and some forms of maltreatment can overlap. For instance, sexual abuse can also involve physical abuse, and all forms of maltreatment include an element of emotional or psychological abuse, which can further complicate the definitions [7]. Current evidence indicates that childhood maltreatment, whether sexual or non-sexual, has significant physical and mental consequences in the short and long term [6,8]. Moreover, exposure to multiple forms of childhood maltreatment further increases the risk of developing mental disorders later in life [6,8]. In the general population, childhood maltreatment has been found in 25–43% [9,10] of individuals and it has been associated with the development of obesity over the life course, higher depression rates and a greater prevalence of eating disorders [11,12,13,14]. Up to 70% of patients living with severe obesity prior to BS report a history of childhood maltreatment [15,16], and they are more likely to report the presence of several psychiatric disorders, including mood and anxiety disorders, substance use disorders and binge eating disorder, and greater depressive symptoms [9,17]. Although there is evidence to support the relationship between abuse history and obesity, the association between abuse history and outcomes after BS is not well established [11,18]. Importantly, pre- and post-operative patients’ mental health can impact their adaptions to post-operative behavioral changes, including post-surgical eating behaviors [19,20,21,22], which, in turn, have been shown to be associated with the short- and long-term weight loss outcomes of BS [23,24]. The primary aim of the current study was to explore the relationship between childhood maltreatment and weight loss outcomes at 6 and 12 months post-BS. Our secondary aim was to assess the associations between childhood maltreatment and changes in depressive symptoms and eating behaviors from pre- to post-surgery.

## 2. Materials and Methods

### 2.1. Design and Participants

The REsearch on Bariatric care for Obesity tReatmeNt (REBORN) study is a longitudinal study that assesses adult patients undergoing BS through an ongoing prospective longitudinal study. Patients who were scheduled for a primary BS at a single university bariatric center were approached to participate in REBORN and completed all assessments on site on the day of their clinic visits. For the purpose of the current study, a retrospective analysis of the prospective data from the REBORN database was performed.

The research institution’s ethics board (the Comité d’éthique de la recherche du CIUSSS-NIM (Centre intégré universitaire de santé et de services sociaux du Nord-de-l’île-de-Montréal)) approved the study protocol (REB# 2015-1176), and informed consent was obtained from all study participants prior to participation. Recruitment and data collection procedures of the study were in place to maintain patient confidentiality, which was detailed in the consent form.

Participants’ inclusion criteria for the REBORN study were being admitted for BS (any type) within the university bariatric center, and the exclusion criteria were having had a previous BS. For the purpose of the current report, participants from the REBORN study who were 18 years old and older and completed all items of the Childhood Trauma Questionnaire (CTQ) at baseline were included.

Between October 2015 and March 2018, 1335 patients were screened for the REBORN study, amongst which 614 were found eligible and interested in participating in the study. Of these, 17 patients were excluded from further analyses as they had incomplete demographic data, and out of the remaining 597 patients, only patients who had completed the full CTQ at baseline (*n* = 111) were included in the current report. Baseline sociodemographic characteristics of those who completed the CTQ (*n* = 111) and those who did not (*n* = 486) indicated no significant differences between these groups (see Table S1).

### 2.2. Assessments

Assessments were conducted at baseline (i.e., within the 6 months prior to their BS) and 6 months (6 M) and 12 months post-surgery (12 M). Measures were completed at all time points, except for the CTQ which was administrated only at baseline. Baseline and 6 M assessments were conducted in person on site on the day of their clinic visits, and 12 M assessments were conducted online or by phone.

#### 2.2.1. Anthropometric Measurements

Weight was measured on a digital medical scale and height was measured by a stadiometer, as well as being self-reported (prior to measurement). These measures were taken at baseline and at 6 M post-surgery; however, only self-reported measures were obtained at 12 M. A sensitivity analysis conducted to validate the accuracy of these self-reported measures in our cohort showed sufficient agreement between the self-reported and the measured anthropometric measures (see Figure S1), as reported elsewhere [25]. Thus, for the purpose of this study, self-reported anthropometrics from all time points were used. BMI was calculated by dividing weight by height squared. Percentages of excess weight loss (% EWL) were calculated as follows: ((pre-operative weight-post-operative weight)/(pre-operative weight − ideal weight)) × 100. Ideal body weight was considered the weight for a BMI of 25 kg/m^2^.

#### 2.2.2. Childhood Maltreatment

The CTQ is a self-rating instrument that retrospectively assesses maltreatment during childhood and adolescence. It consists of 28 items, 25 of which are attributed to 5 sub-scales of childhood maltreatment: sexual abuse (SA); physical abuse (PA); emotional abuse (EA); emotional neglect (EN); and physical neglect (PN). An additional three items measure the minimization/denial of the trauma [11,26,27]. The CTQ asks respondents to rate their history of abuse or neglect across these 5 sub-scales, using a 5-point Likert-like scale with values ranging from “Never True” to “Very Often True” [11,26,27,28]. The scoring of each sub-dimension is from 5 to 25, and total CTQ scoring ranges from 25 to 125 [11,26,27]. Thresholds to define the presence and severity for each sub-scale score have been previously described (i.e., none (or minimal), low, moderate and severe) [17,29]. In addition, for each category of abuse, participants with scores in the minimal range were classified as negative for exposure, and participants with scores in the low–severe range were classified as positive for exposure [17,29]. Previously reported internal consistency for the CTQ total and sub-scales were very good, ranging between Cronbach’s alpha (α) coefficient = 0.82 and 0.90 [11,28]. In the current sample, the CTQ total score demonstrated excellent internal consistency (α = 0.92), while α coefficients of the CTQ sub-scales were α = 0.85, α = 0.97, α = 0.45, α = 0.82 and α = 0.91 for the EA, SA, PN, PA and EN sub-scale scores, respectively.

#### 2.2.3. Depressive Symptoms

The Beck Depression Inventory-II (BDI-II) is a 21-item self-report questionnaire that measures depressive symptomatology, in which patients rate the severity of depressive symptoms during the previous 2 weeks on a 4-point scale ranging from 0 to 3 [30,31]. Total summed score ranging from 0 to 13 indicates minimal depressive symptoms, 14 to 19 indicates mild depressive symptoms, 20 to 28 indicates moderate depressive symptoms and 29 to 63 indicates severe depressive symptoms [30,31]. Internal consistency for the total scale score of this instrument has been well established [30,32]; moreover, this questionnaire is frequently used to assess changes in depressive symptoms following BS [11,28,33]. Internal consistency within our sample for the total BDI-II score was high (α = 0.87).

#### 2.2.4. Eating Behavior

The Dutch Eating Behavior Questionnaire (DEBQ) evaluates emotional eating (EE), external eating (ExE) and restrained eating (RE) behaviors and is composed of 33 items that are evaluated by a 5-point Likert-like scale (ranging from never to very often) [34,35]. The RE scale measures intentions to restrict food intake and control of food intake. EE corresponds to the tendency to overeat in response to negative emotions. ExE corresponds to the tendency to overeat in response to food-related stimuli [36]. Items on the sub-scales are averaged, with higher mean scores indicative of greater restrained eating, eating in response to emotions and eating triggered by external cues [33,34,35]. Consistent with other studies [33,36], the DEBQ total score demonstrated excellent internal consistency (α = 0.90), while the sub-scales ranged between moderate (EE, α = 0.76) and excellent internal consistency (EE, α = 0.96).

### 2.3. Statistical Analysis

Statistical analyses were conducted using both SPSS and R statistical (ggplot package) programs. The significance threshold was *p* < 0.05 for all analyses. If normality was rejected by the Kolmogorov–Smirnov test, non-parametric tests were used. Continuous variables are presented as means ± standard deviation (SD) or median and interquartile range (IQR), as appropriate, and dichotomous/categorical variables as proportions. In a first step, descriptive statistics at baseline were calculated to characterize the sample in terms of sociodemographic characteristics as well as anthropometrics, eating behaviors, depressive symptoms and prevalence rates of different forms of childhood maltreatment. Comparisons between groups of their continuous variables were carried out using either the independent-samples *t*-test or the Mann–Whitney test, while comparisons between dichotomous or categorical variables were assessed using the Pearson chi-square (*x*^2^) test. The impact of childhood maltreatment on the course of weight loss outcomes, eating behaviors and depressive symptom scores at 6 M and 12 M after BS was analyzed with linear mixed models with repeated measures. Post-operative BMI, %EWL, eating behaviors and depressive symptom scores were included as dependent variables and time (baseline (pre-surgery), 6 M follow-up, 12 M follow-up) and childhood maltreatment (yes vs. no for any type of trauma of the five trauma sub-scales) as fixed factors. These models were adjusted for sociodemographic variables (sex, age, education level), surgery type and baseline BMI.

## 3. Results

### 3.1. Sample Characteristics

Patients within the final study sample (*n* = 111) underwent either sleeve gastrectomy (SG) (*n* = 98), Roux-en-Y gastric bypass (RYGB) (*n* = 10) or single anastomosis duodeno-ileal bypass with sleeve gastrectomy (SADI) (*n* = 3). The mean age and median BMI of participants were 45.1 ± 11.7 years and 46.7 (IQR of 42.4–51.9) kg/m^2^, respectively, and this sample included 84.7% (*n* = 94) females. Baseline characteristics of the total sample and according to sex are reported in Table 1 and Table S2***,*** respectively. Prior to BS, females had lower rates of type 2 diabetes, hypercholesterolemia and reduced BMI compared to males and higher scores on the emotional eating and CTQ sexual abuse sub-scales (Table S2).

### 3.2. History of Childhood Maltreatment among Patients Prior to BS

**Figure 1:**
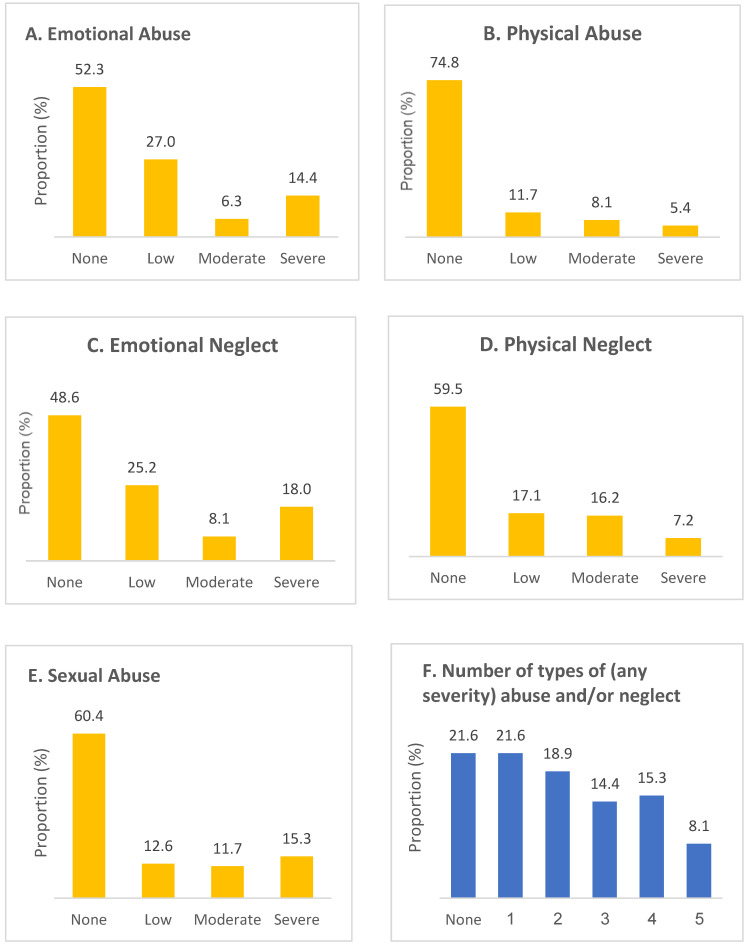
Prevalence of childhood maltreatment history (emotional abuse (**A**), physical abuse (**B**), emotional neglect (**C**), physical neglect (**D**) and sexual abuse (**E**) and number of maltreatments of any type of any severity (**F**)) in the sample (*n* = 111).

Histories of EA, PA, EN, PN and SA were reported by 47.7%, 25.2%, 51.4%, 40.5% and 39.6% of the patients, respectively (Figure 1). More than three-quarters (78.4%) of patients reported at least one form of abuse or neglect. Having a history of one, two, three, four and five types of traumas was reported among 21.6%, 18.9%, 14.4%, 15.3% and 8.1% of participants, respectively (Figure 1F). The baseline characteristics across patients with and without a history of experiencing each type of maltreatment are presented in Table 2a,b. Patients with a history of EA had higher baseline BMI and depressive symptom scores compared to those without a history of this type of abuse. Higher baseline depressive symptom scores were also observed among patients with a history of EN and PA compared to those without a history. Additionally, patients with a history of SA and EN had higher baseline scores on the EE and ExE sub-scales of the DEBQ compared to patients without, respectively (Table 2a,b).

### 3.3. Changes in Weight

Changes in anthropometric measurements during the study period according to exposure to different maltreatment types are presented in Table 3. There was a significant main effect for time regarding both BMI and %EWL, with significant gradual weight loss being achieved by 12 M post-surgery. However, there was no significant main effect of group, which indicated that changes in BMI and %EWL did not differ between patients with or without a history of exposure to maltreatment (regardless of type), and no significant interactions were found between time and group for BMI and %EWL outcomes across all maltreatment history types (Table 3).

### 3.4. Changes in Depressive Symptoms

**Figure 2:**
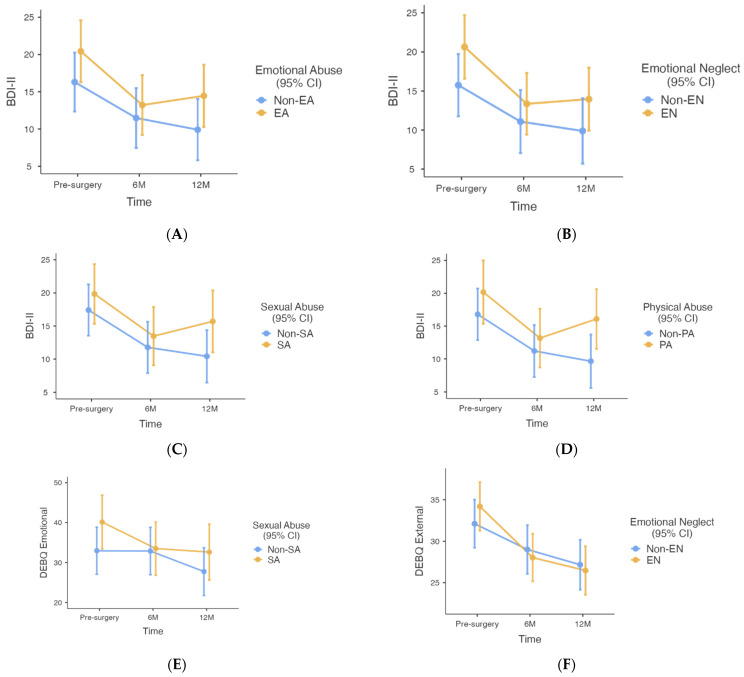
Course of depressive symptoms based on the BDI-II total scores (**A**–**D**) and eating behaviors based on the DEBQ and its sub-scale scores (**E**,**F**) according to the status of maltreatment type. Abbreviations: Beck Depression Inventory-II (BDI-II), Dutch Eating Behavior Questionnaire (DEBQ), 6 and 12 months post-operation (6 M, 12 M), emotional abuse (EA), emotional neglect (EN), physical neglect (PA), sexual abuse (SA). Data were available for 111, 101 and 73 participants at baseline, 6 M and 12 M, accordingly.

Changes in depressive symptoms by different maltreatment types are presented in Table 3 and Figure 2. There was a main effect of time, demonstrated by the decreased BDI-II scores from pre- to post-surgery. We found a significant main effect of history of exposure to all types of maltreatment (except for PN) on depressive symptom scores, which were demonstrated to be higher at all time points among participants reporting a history of maltreatment. However, there were no interactions between childhood maltreatment and time (Table 3, Figure 2). 

### 3.5. Changes in Eating Behaviors

All three eating behavior sub-scale scores on the DEBQ (EE, ExE and RE behaviors) decreased significantly over time from pre- to post-surgery for all patients (Table 3, Figure 2). There were two statistically significant interactions: one between time and a history of SA on EE and one between time and history of EN on ExE behaviors. Specifically, EE scores decreased 6 M post-surgery among patients with a history of SA, but no further score reductions were observed at 12 M post-surgery. In contrast, while EE scores among those without a history of SA remained stable during the early post-surgery period (6 M), they had significantly improved by 12 M post-operation. Despite demonstrating greater improvements in ExE during the early post-operative period (i.e., from pre-surgery to 6 M post-surgery), patients with a history of EN showed comparable ExE scores to those without EN at 12 M post-surgery (Table 3, Figure 2).

## 4. Discussion

This study is among the few that have simultaneously evaluated the prospective impact of childhood maltreatment on three important BS outcomes, i.e., changes in weight, depressive symptoms and eating behaviors from pre- to post-surgery. The high number of patients who experienced at least one form of abuse or neglect in our sample (nearly 80% of patients) is quite striking. Nonetheless, previous reports in bariatric candidates living with severe obesity ranged from 66–70% [9,17,37], which is significantly higher compared to the rates observed in the general population and normative community samples (25–43%) [9,10,38]. Our results elaborate upon previous findings regarding the prevalence of specific types of childhood maltreatment among patients pre-BS [18], as we found a higher prevalence of PN and EN history within our sample (40.5% and 51.4%), compared to ranges described by previous literature in this population (17.4 to 32.1% and 39.0 to 48.8%, respectively) [18].

Despite being related to changes in eating behaviors, a history of childhood maltreatment was not associated with post-surgery weight outcomes. While it is difficult to compare across available studies due to the variability in methods and study populations, our results add to the previous literature, showing that childhood maltreatment may not impact weight loss after BS [18], at least up to 12 months.

Although weight loss is the most commonly reported BS outcome, it is important to consider other post-operative outcomes that may directly or indirectly influence weight changes post-BS. Several studies to date have found significant relationships between a history of abuse and neglect and non-weight-related outcomes, including eating psychopathology, depressive symptoms and even inpatient hospitalizations [18]. Our results indicate a different course of emotional eating post-surgery in patients with versus without a history of SA. Although this eating behavior seems to decrease more in the early post-operative stage among those with prior exposure to SA compared to those without, the latter eventually experienced greater improvements 12 months post-operatively, having overall lower EE scores by the end of the post-operative follow-up period. Moreover, despite demonstrating greater improvements in ExE during the early (6-month) post-operative period, patients with a history of EN showed comparable ExE scores to those without EN at 12 M post-BS. Improvements in some eating pathologies were previously observed mainly during the early post-operative period across different types of BS, likely due to the anatomically restricted capacity to binge or overeat that is imposed by the surgical procedure [39,40,41,42,43]. However, at longer-term follow-ups, maladaptive eating may revert and even return to pre-surgery levels [40,43]. Thus, it is possible that both emotional and external eating behaviors in patients with a history of maltreatment were “prevented” in the early post-surgical period due to these physical restrictions, rather than due to any volitional changes in eating behavior. The fact that eating behavior scores were comparable or higher among those with a history of maltreatment compared to those without at 12 M provides partial support for this hypothesis. Comparisons to previous literature on this matter are challenging given the limited number of studies assessing the impact of childhood maltreatment on post-operative eating behaviors in this population, as well as variability across studies in their assessment of childhood maltreatment and eating behaviors [9,11,18,28]. Nevertheless, in contrast to the low number of studies that focused on such post-surgical changes, the nature of the relationship between childhood maltreatment and problematic eating behaviors prior to BS and among patients living with severe obesity has been investigated more extensively [35,44]. Indeed, higher CTQ scores have been associated with increased binge eating, food addiction and emotional eating behaviors [35,44,45]. Previous studies also provide some indications that emotional and physical abuse history are predictors of increased EE in this population [35,44,45]. Taken together with previous associations between certain problematic eating behaviors and increased BMI pre- and post-surgery, these findings reinforce the importance of EE among those with exposure to childhood maltreatment who then go on to develop severe obesity requiring bariatric surgery [35,44,45].

Unfortunately, we were limited by the sample size and the relatively short length of follow-up to be able to test some more advanced associations (e.g., mediation/moderation models) that could have shed light on the nature of the relationships between varied factors, i.e., eating behaviors, depression course and weight measures, in relation to a history of childhood maltreatment. Nevertheless, taken together with our findings, the available literature suggests that there may be differences between the type of maltreatment and the dynamics of problematic eating behaviors and their associations with pre- and post-operative weight and mental health status. This highlights a need for future studies with larger sample sizes and longer follow-ups to disentangle the mechanisms linking childhood maltreatment to obesity [18,35] and how these are impacted by BS. Collectively, eating behaviors may be one of the factors involved within the pathway both towards severe obesity and post-surgical weight outcomes, where they have been shown to be intermediary maladaptive coping mechanisms to childhood abuse or neglect, which have then been associated with greater weight gain [46].

In the current study, depressive symptom scores were demonstrated to be higher at all time points among participants exposed to all types of maltreatment except for PN. The results of other studies that examined the association of childhood maltreatment with post-operative depressive symptoms are mixed [18], but a notable number of studies indicate a significant relationship between a history of maltreatment and increased levels of depressive symptoms after BS. Lu et al. followed participants for 6 months and found that mean depressive symptom scores were significantly higher in the group with a history of PA and SA compared to those without [47], and Larsen et al. found that patients with a history of SA had significantly higher depressive symptom scores at 34 months post-BS compared to patients without [48]. However, other studies found no association between childhood maltreatment history and depression after BS [11,18].

## 5. Limitations and Strengths

This study had some limitations, including evaluation by self-report measures without a psychiatric interview and the lack of data on psychiatric co-morbidities and other potentially important eating disorder diagnoses (e.g., binge eating). Moreover, this study was not able to assess moderation and/or mediation models or undertake potential sub-group analyses to examine the interactions between different factors that could explain the relationships and process between childhood maltreatment, psychological factors and BS outcomes. Nevertheless, the current study had a number of notable strengths. First, it included validated measures of childhood maltreatment, depressive symptoms and eating behaviors. With the exception of the PN scale, the internal consistency of all other instruments used and their sub-scales within our sample was robust. A second strength is the prospective nature of this study design and a follow-up that extended to 12 M post-surgery. Third, the majority of published research on childhood maltreatment and BS has focused on the pre-surgical period, and on SA specifically, and as such, this study adds to the extant literature by focusing on several types of maltreatment. Fourth, in contrast to previous studies in this field, we were able to adjust for some important confounding factors (e.g., surgery type, sex, age, etc.) in our multivariate models. Finally, though we had a relatively small sample who completed the CTQ, there did not seem to be a selection bias with our cohort (see Table S1 for comparisons between our sample and the larger bariatric cohort it was derived from).

## 6. Conclusions

In conclusion, our results indicate that childhood maltreatment does not have a significant effect on 12-month weight changes after BS. However, given the extremely high prevalence of childhood maltreatment experienced by bariatric patients and their associations with both pre- and post-BS eating behaviors and depressive symptoms, a history of maltreatment may have important clinical implications, including playing a role in the etiology of dysfunctional eating behavior, which may reflect a maladaptive coping mechanism for abuse [49].

### 6.1. Practical Clinical Implications

The inclusion of a broader set of psychological assessments, continued monitoring and the development of psychological interventions seem essential to support patients’ adaptive recovery from abuse and the adoption of healthier eating patterns after surgery.

### 6.2. Future Research Implications

Future prospective studies with larger sample sizes and longer follow-ups are needed to disentangle the mechanisms linking childhood maltreatment to obesity and how these are impacted by BS.

## Figures and Tables

**Table 1:** Baseline characteristics of the study sample (*n* = 111).

Parameter †	Total Sample (*n* = 111)
Sociodemographic and medical background (*n* = 111)	
Sex (*n* (% females))	94 (84.7)
Age (years)	45.1 (11.7)
Race (*n* (% White))	99 (89.2)
Education (years of studies)	14.0 (11.0–16.0)
Marital status	
Single (*n* (%))	30 (27.0)
Common-law partner (*n* (%))	28 (25.2)
Married (*n* (%))	44 (39.6)
Divorced/separated (*n* (%))	9 (8.1)
Widowed (*n* (%))	0 (0.0)
Monthly income	
Less than CAD 23,000 (*n* (%))	12 (10.9)
CAD 23,001–37,000 (*n* (%))	11 (10.0)
CAD 37,001–57,000 (*n* (%))	25 (22.7)
CAD 57,001–84,000 (*n* (%))	23 (20.9)
More than CAD 84,000 (*n* (%))	29 (26.4)
Don’t know/not answered (*n* (%))	11 (9.9)
Type 2 diabetes (*n* (%))	25 (22.5)
Hypertension (*n* (%))	45 (40.5)
High LDL cholesterol (*n* (%))	29 (26.1)
Currently seeing psychologist/psychiatrist (*n* (%))	29 (26.1)
Weight (kg)	129.0 (110.0–144.0)
BMI (kg/m^2^)	46.7 (42.4–51.9)
Smoking status (*n*, (%yes))	11 (9.9)
History of childhood maltreatment based on CTQ scores (*n* = 111)	
Total CTQ score	38.0 (31.0–54.5)
Emotional abuse (scale score)	8.0 (6.0–12.0)
Physical abuse (scale score)	5.0 (5.0–7.5)
Emotional neglect (scale score)	10.0 (7–15)
Physical neglect (scale score)	7.0 (5.0–9.0)
Sexual abuse (scale score)	5.0 (5.0–8.0)
Eating behavior based on DEBQ scores (*n* = 79)	
DEBQ restrained eating scale	29.0 (24.0–31.5)
DEBQ emotional eating scale	39.0 (12.9)
DEBQ external eating scale	24.0 (5.3)
Depressive symptoms based on BDI-II scores (*n* = 85)	
BDI-II total score	14.0 (8.0–21.0)
^1^ Symptomology classifications	
Minimal depression (*n* (%))	39 (45.9)
Mild depression (*n* (%))	21 (24.7)
Moderate depression (*n* (%))	16 (18.8)
Severe depression (*n* (%))	9 (10.6)

† Values are expressed as the mean (SD) or median (25th–75th percentile) according to the variable. Abbreviations: BDI-II, Beck Depression Inventory-II; BMI, body mass index (BMI); CTQ, Childhood Trauma Questionnaire; DEBQ, Dutch Eating Behavior Questionnaire; LDL, low-density lipoprotein. ^1^ Symptomology classifications were based on the BDI-II total summed scores: score range of 0 to 13 indicates minimal depressive symptoms; 14 to 19 indicates mild depressive symptoms; 20 to 28 indicates moderate depressive symptoms; 29 to 63 indicates severe depressive symptoms.

**Table 2:** (**a**) Baseline characteristics by the status of different maltreatment types (emotional abuse, physical abuse and emotional neglect) among the total sample. (**b**) Baseline characteristics by the status of different maltreatment types (physical neglect and sexual abuse) among the total sample.

**(a)**												
**Parameter †**	**Non-EA** ***n* = 58**	**EA** ***n* = 53**	***p*-Value**	**Non-PA** ***n* = 83**		**PA** ***n* = 28**		***p*-Value**	**Non-EN** ***n* = 54**	**EN** ***n* = 57**		***p*-Value**
Age (years)	45.4 (11.7)	44.9 (11.7)	0.849	44.0 (12.1)		48.5 (9.8)		0.078	45.7 (11.7)	44.6 (44.7)		0.601
Education (years of studies)	14.0 (11.0–16.0)	14.0 (11.0–15.0)	0.445	14.0 (11.0–16.0)		13.5 (11.0–16.0)		0.880	14.0 (11.0–15.0)	14.0 (11.0–16.0)		0.922
Marital status Single (*n* (%)) Common-law partner (*n* (%)) Married (*n* (%)) Divorced/separated (*n* (%)) Widowed (*n* (%))	15 (25.9) 13 (22.4) 26 (44.8) 4 (6.9) 0 (0.0)	15 (2.8) 15 (2.8) 18 (34.0) 5 (9.4) 0 (0.0)	0.685	25 (30.1) 21 (25.3) 31 (37.3) 6 (7.2) 0 (0.0)		5 (17.9) 7 (25.0) 13 (46.4) 3 (10.7) 0 (0.0)		0.590	12 (22.2) 14 (25.9) 23 (42.6) 5 (9.3) 0 (0.0)	18 (31.6) 14 (24.6) 21 (36.8) 4 (7.0) 0 (0.0)		0.724
Monthly income Less than CAD 23,000 (*n* (%)) CAD 23,001–37,000 (*n* (%)) CAD 37,001–57,000 (*n* (%)) CAD 57,001–84,000 (*n* (%)) More than CAD 84,000 (*n* (%)) DK/not answered (*n* (%))	5 (8.6) 7 (12.1) 10 (17.2) 15 (25.9) 14 (24.1) 7 (12.1)	7 (13.2) 4 (7.5) 15 (28.3) 8 (15.1) 15 (28.3) 4 (7.5)	0.244	10 (12.0) 8 (9.6) 18 (21.7) 19 (22.9) 21 (25.3) 7 (8.4)		2 (7.1) 3 (10.7) 7 (25.0) 4 (14.3) 8 (28.6) 4 (14.3)		0.954	6 (11.1) 6 (11.1) 10 (18.5) 15 (27.8) 14 (25.9) 3 (5.6)	6 (10.7) 5 (8.9) 15 (26.8) 8 (14.3) 15 (26.8) 8 (14.0)		0.564
Weight (kg)	123.2 (109.2–140.6)	132.0 (116.6–148.3)	0.035	130.3 (115.2–144.5)		121.6 (108.9–144.0)		0.356	124.0 (107.0–143.0)	132 (119.0–145.0)		0.084
BMI (kg/m^2^)	45.2 (42.4–49.5)	48.8 (43.5–53.7)	0.023	46.9 (42.6–51.9)		45.7 (41.2–45.7)		0.658	45.8 (42.1–49.6)	48.6 (43.4–52.8)		0.081
Smoking status (*n*, (yes%))	4 (6.9)	7 (13.2)	0.266	7 (8.4)		4 (14.3)		0.370	5 (9.3)	6 (10.5)		0.823
^2^ DEBQ restrained eating	29.0 (24.5–30.0)	29.0 (24.0–34.3)	0.362	29.0 (24.5–31.5)		28.0 (23.8–32.5)		0.972	29.0 (25.0–30.0)	28.0 (23.8–34.0)		0.739
^2^ DEBQ emotional eating	37.8 (13.5)	40.6 (12.2)	0.342	39.2 (12.8)		38.1 (13.8)		0.742	37.0 (12.0)	41.2 (13.8)		0.150
^2^ DEBQ external eating	31.5 (4.9)	32.4 (5.9)	0.461	32.2 (5.4)		30.7 (5.2)		0.312	30.8 (4.8)	33.1 (5.7)		0.036
^1^ BDI-II total score	11.0 (7.0–17.0)	17.0 (10.8–25.3)	0.012	13.0 (8.0–20.0)		19.0 (11.8–22.5)		0.224	10.0 (6.0–17.0)	18.0 (13.8–24.3)		0.002
^1,3^ Depression classification ^1,3^ Minimal (*n* (%)) ^1,3^ Mild (*n* (%)) ^1,3^ Moderate (*n* (%)) ^1,3^ Severe (*n* (%))	28 (57.1) 12 (24.5) 6 (12.2) 3 (6.1)	11 (30.6) 9 (25.0) 10 (27.8) 6 (16.7)	0.017	35 (50.7) 16 (23.2) 12 (17.4) 6 (8.7)		4 (25.0) 5 (31.3) 4 (25.0) 3 (18.8)		0.026	29 (64.4) 7 (15.6) 6 (13.3) 3 (6.7)	10 (25.0) 14 (35.0) 10 (25.0) 6 (15.0)		0.004
**(b)**												
**Parameter †**	**Non-PN** ***n* = 66**		**PN** ***n* = 45**		***p*-Value**		**Non-SA** ***n* = 67**		**SA** ***n* = 44**		***p*-Value**	
Age (years)	45.6 (11.3)		44.4 (12.3)		0.602		44.2 (10.9)		46.5 (12.7)		0.314	
Education (years of studies)	14.0 (12.0–16.0)		13.0 (11.0–15.0)		0.104		14.0 (12.0–16.0)		13.0 (11.0–15.0)		0.057	
Marital status Single (*n* (%)) Common-law partner (*n* (%)) Married (*n* (%)) Divorced/separated (*n* (%)) Widowed (*n* (%))	18 (27.2) 20 (30.3) 24 (36.4) 4 (6.1) 0 (0.0)		12 (26.7) 8 (17.8) 20 (44.4) 5 (11.1) 0 (0.0)		0.399		17 (25.4) 13 (19.4) 31 (46.3) 6 (9.0) 0 (0.0)		13 (29.5) 15 (34.1) 13 (29.5) 3 (6.8) 0 (0.0)		0.215	
Monthly income Less than CAD 23 000 (*n* (%)) CAD 23 001–37 000 (*n* (%)) CAD 37 001–57 000 (*n* (%)) CAD 57 001–84 000 (*n* (%)) More than CAD 84 000 (*n* (%)) DK/not answered (*n* (%))	8 (12.1) 6 (9.1) 13 (19.7) 15 (22.7) 20 (30.3) 4 (6.1)		4 (8.9) 5 (11.1) 12 (26.7) 8 (17.8) 9 (20.0) 7 (15.6)		0.429		6 (9.1) 7 (10.6) 11 (16.7) 12 (18.2) 23 (34.8) 8 (11.9)		6 (13.6) 4 (9.1) 14 (31.8) 11 (25.0) 6 (13.6) 3 (6.8)		0.188	
Weight (kg)	124.5 (109.7–144.4)		131.5 (118.8–144.2)		0.385		131.0 (115.0–149.0)		126.0 (109.0–136.0)		0.112	
BMI (kg/m^2^)	45.8 (42.4–50.8)		48.8 (42.4–52.7)		0.246		46.8 (42.4–52.8)		46.5 (42.3–50.3)		0.544	
Smoking status (*n*, (yes%))	5 (7.6)		6 (13.3)		0.319		8 (11.9)		3 (6.8)		0.377	
^2^ DEBQ restrained eating	29.0 (26.0–31.0)		26.5 (22.3–32.3)		0.183		28.0 (24.0–30.0)		30.0 (25.0–34.0)		0.251	
^2^ DEBQ emotional eating	37.8 (12.7)		41.1 (13.4)		0.264		35.7 (12.8)		44.1 (11.7)		*0.004*	
^2^ DEBQ external eating	31.4 (5.8)		32.7 (4.4)		0.269		31.3 (5.3)		32.9 (5.2)		0.173	
^1^ BDI-II total score	12.0 (6.25–21.0)		15.0 (10.5–20.0)		0.128		13.0 (8.0–19.0)		16.0 (10.0–24.3)		0.226	
^1,3^ Depression classification ^1,3^ Minimal (*n* (%)) ^1,3^ Mild (*n* (%)) ^1,3^ Moderate (*n* (%)) ^1,3^ Severe (*n* (%))	28 (51.2) 10 (18.5) 12 (22.2) 4 (7.4)		11 (35.5) 11 (35.5) 4 (12.9) 5 (16.1)		0.090		27 (50.9) 14 (26.4) 8 (15.1) 4 (7.5)		12 (37.5) 7 (21.9) 8 (25.0) 5 (15.6)		0.359	

† Values are expressed as the mean (SD) or median (25th–75th percentile) according to the variable. Abbreviations: BDI-II, Beck Depression Inventory-II; BMI, body mass index (BMI); CTQ, Childhood Trauma Questionnaire; DEBQ, Dutch Eating Behavior Questionnaire; DK, don’t know; EA, emotional abuse; EN, emotional neglect; PA, physical abuse; ^1^ BDI-II scores were available for 85 participants at baseline; ^2^ DEBQ scores were available for 79 participants at baseline. ^3^ Symptomology classifications were based on the BDI-II total summed scores: score range of 0 to 13 indicates minimal depressive symptoms; 14 to 19 indicates mild depressive symptoms; 20 to 28 indicates moderate depressive symptoms; 29 to 63 indicates severe depressive symptoms.

**Table 3:** Changes in weight loss, depressive scores and eating behaviors during the study period according to the status of maltreatment types.

^1^ Outcome Variable †	Group	Baseline Mean (SE) [95% CI]	6 M Mean (SE) [95% CI]	12 M Mean (SE) [95% CI]	Main Effect Time		Main Effect Group		Time×Group	
					*p*	F	*p*	F	*p*	F
Non-EA (*n* = 58) compared to EA (*n* = 53) based on CTQ at baseline										
BMI (kg/m^2^)	Non-EA	46.1 (1.6) [43.1–49.2]	34.2 (1.6) [31.1–37.3]	31.9 (1.6) [28.8–35.1]	<0.001	528.70	0.083	3.07	0.105	2.28
	EA	49.2 (1.5) [46.2–52.3]	35.5 (1.5) [32.5–38.6]	33.2 (1.6) [30.1–36.4]						
EWL (%)	Non-EA	-	60.2 (5.6) [49.1–71.3]	70.7 (5.7) [59.5–82.0]	<0.001	35.00	0.820	0.05	0.808	0.06
	EA		60.7 (5.5) [49.8–71.6]	72.1 (5.7) [60.8–83.4]						
Total BDI-II score	Non-EA	16.3 (2.0) [12.3–20.3]	11.5 (2.0) [7.5–15.5]	9.9 (2.0) [5.8–14.0]	<0.001	32.33	0.018	5.81	0.219	1.53
	EA	20.4 (2.1) [16.3–24.6]	13.2 (2.0) [9.2–17.2]	14.5 (2.1) [10.3–18.6]						
DEBQ restrained eating	Non-EA	27.6 (2.0) [23.6–31.5]	32.3 (2.0) [28.2–36.3]	31.9 (2.1) [27.7–36.1]	0.003	6.27	0.492	0.47	0.090	2.46
	EA	29.1 (2.1) [24.9–33.3]	31.0 (2.1) [26.9–35.2]	28.8 (2.3) [24.3–33.3]						
DEBQ emotional eating	Non-EA	35.4 (3.1) [29.2–41.6]	34.2 (3.2) [28.0–40.5]	29.0 (3.2) [22.6–35.3]	<0.001	11.65	0.718	0.13	0.317	1.16
	EA	35.5 (3.2) [29.1–42.0]	31.2 (3.2) [24.9–37.5]	29.4 (3.3) [22.9–35.8]						
DEBQ external eating	Non-EA	32.9 (1.4) [30.1–35.8]	29.4 (1.5) [26.5–32.3]	27.3 (1.5) [24.3–30.2]	<0.001	59.43	0.341	0.91	0.130	2.07
	EA	33.1 (1.5) [30.2–36.1]	27.3 (1.5) [24.5–30.2]	26.2 (1.5) [23.2–29.1]						
Non-EN (*n* = 54) compared to EN (*n* = 57) based on CTQ at baseline										
BMI (kg/m^2^)	Non-EN	46.8 (1.6) [43.6–49.9]	34.8 (1.6) [31.6–37.9]	32.4 (1.6) [29.2–35.6]	<0.001	523.73	0.440	0.60	0.202	1.62
	EN	48.6 (1.6) [45.5–51.7]	35.0 (1.6) [31.2–38.1]	32.9 (1.6) [29.7–36.0]						
EWL (%)	Non-EN	-	59.9 (5.6) [48.7–71.1]	72.2 (5.8) [60.8–83.6]	<0.001	34.76	0.971	0.01	0.448	0.58
	EN		61.2 (5.5) [50.3–72.0]	70.7 (5.6) [59.9–81.7]						
Total BDI-II score	Non-EN	15.7 (2.0) [11.8–19.7]	11.1 (2.0) [7.0–15.1]	9.9 (2.0) [5.7–14.1]	<0.001	33.07	0.009	7.08	0.283	1.28
	EN	20.6 (2.0) [16.6–24.7]	13.4 (2.0) [9.4–17.3]	14.0 (2.0) [9.9–18.0]						
DEBQ restrained eating	Non-EN	28.2 (2.0) [24.0–31.9]	32.4 (2.1) [28.3–36.5]	31.2 (2.2) [26.9–35.5]	0.001	6.92	0.576	0.32	0.536	0.63
	EN	28.4 (2.1) [24.2–32.6]	30.9 (2.1) [26.8–35.0]	30.0 (2.2) [25.6–34.3]						
DEBQ emotional eating	Non-EN	34.5 (3.2) [28.2–40.8]	34.1 (3.2) [27.7–40.5]	29.3 (3.3) [22.8–35.9]	<0.001	12.46	0.975	0.00	0.169	1.80
	EN	36.7 (3.2) [30.4–43.1]	31.8 (3.1) [25.6–38.0]	29.1 (3.2) [22.8–35.4]						
DEBQ external eating	Non-EN	32.1 (1.5) [29.2–35.0]	29.0 (1.5) [26.1–31.9]	27.2 (1.5) [24.1–30.2]	<0.001	63.33	0.895	0.02	0.013	4.52
	EN	34.2 (1.5) [31.3–37.1]	28.0 (1.4) [25.2–30.9]	26.5 (1.5) [23.5–29.4]						
Non-SA (*n* = 67) compared to SA (*n* = 44) based on CTQ at baseline										
BMI (kg/m^2^)	Non-SA	47.9 (1.5) [45.0–50.9]	35.0 (1.5) [32.0–38.0]	32.3 (1.5) [29.3–35.4]	<0.001	467.86	0.969	0.01	0.311	1.18
	SA	47.2 (1.7) [43.8–50.5]	34.7 (1.7) [31.3–38.0]	33.3 (1.8) [29.8–36.8]						
EWL (%)	Non-SA	-	59.6 (5.3) [49.1–70.1]	72.6 (5.4) [62.0–83.3]	<0.001	26.62	0.962	0.00	0.120	2.47
	SA		62.5 (6.0) [50.5–74.4]	69.4 (6.2) [57.0–81.8]						
Total BDI-II score	Non-SA	17.4 (2.0) [13.5–21.3]	11.8 (2.0) [7.9–15.7]	10.4 (2.0) [16.5–14.4]	<0.001	28.93	0.049	3.96	0.165	1.83
	SA	19.8 (2.3) [15.3–24.3]	13.5 (2.0) [9.1–17.9]	15.7 (2.4) [11.0–20.4]						
DEBQ restrained eating	Non-SA	27.5 (2.0) [23.6–31.4]	31.4 (2.0) [27.5–35.3]	31.0 (2.1) [26.9–35.1]	0.004	5.75	0.685	0.17	0.423	0.87
	SA	29.5 (2.3) [25.0–34.0]	32.2 (2.2) [27.8–36.6]	30.0 (2.4) [25.2–34.9]						
DEBQ emotional eating	Non-SA	33.0 (3.0) [27.1–38.9]	32.9 (3.0) [27.1–38.8]	27.8 (3.0) [21.8–33.7]	<0.001	11.78	0.083	3.07	0.026	3.76
	SA	40.1 (3.4) [33.4–46.9]	33.5 (3.4) [26.9–40.2]	32.6 (3.5) [25.6–39.6]						
DEBQ external eating	Non-SA	32.4 (1.4) [29.6–35.1]	28.5 (1.4) [25.7–31.3]	26.3 (1.4) [23.5–29.1]	<0.001	54.49	0.410	0.69	0.235	1.46
	SA	34.0 (1.6) [30.8–37.2]	28.3 (1.6) [25.2–31.4]	27.7 (1.7) [24.3–31.0]						
Non-PN (*n* = 66) compared to PN (*n* = 45) based on CTQ at baseline										
BMI (kg/m^2^)	Non-PN	47.7 (1.6) [44.6–50.9]	35.4 (1.6) [32.2–38.6]	33.1 (1.6) [29.9–36.3]	<0.001	494.19	0.615	0.25	0.457	0.79
	PN	47.9 (1.6) [44.8–51.0]	34.4 (1.6) [31.3–37.5]	32.2 (1.6) [28.9–35.4]						
EWL (%)	Non-PN	-	57.6 (5.6) [46.5–68.7]	69.6 (5.7) [61.2–84.0]	<0.001	29.54	0.294	1.11	0.463	0.55
	PN		63.5 (5.6) [52.5–74.5]	72.6 (5.7) [61.2–84.0]						
Total BDI-II score	Non-PN	17.6 (2.0) [13.5–21.7]	11.5 (2.0) [7.4–15.7]	11.3 (2.0) [7.1–15.5]	<0.001	26.13	0.402	0.71	0.956	0.05
	PN	18.5 (2.0) [14.2–22.8]	13.0 (2.0) [8.8–17.2]	12.8 (2.0) [8.3–17.2]						
DEBQ restrained eating	Non-PN	28.9 (2.0) [24.8–32.9]	32.5 (2.1) [28.4–36.6]	32.1 (2.2) [27.9–36.4]	0.003	5.98	0.185	1.78	0.432	0.84
	PN	28.1 (2.2) [23.9–32.4]	31.3 (2.1) [27.2–35.3]	28.5 (2.3) [23.9–33.0]						
DEBQ emotional eating	Non-PN	34.3 (3.2) [28.0–40.5]	31.5 (3.2) [25.2–37.8]	28.2 (3.2) [21.8–34.6]	<0.001	10.14	0.396	0.73	0.946	0.06
	PN	36.4 (3.3) [29.9–42.9]	33.9 (3.2) [27.6–40.2]	29.7 (3.4) [23.0–36.4]						
DEBQ external eating	Non-PN	32.8 (1.5) [29.9–35.7]	28.8 (1.5) [25.8–31.7]	26.9 (1.5) [23.9–29.8]	<0.001	53.67	0.853	0.03	0.443	0.81
	PN	33.4 (1.5) [30.4–36.4]	27.9 (1.5) [25.0–31.8]	26.5 (1.6) [23.4–29.7]						
Non-PA (*n* = 83) compared to PA (*n* = 28) based on CTQ at baseline										
BMI (kg/m^2^)	Non-PA	47.7 (1.6) [44.7–50.8]	35.1 (1.6) [32.0–38.2]	32.7 (1.6) [29.6–35.8]	<0.001	403.01	0.867	0.03	0.858	0.15
	PA	47.7 (1.7) [44.4–51.1]	34.5 (1.7) [31.1–37.9]	32.7 (1.7) [29.2–36.1]						
EWL (%)	Non-PA	-	59.3 (5.4) [48.5–70.1]	70.7 (5.6) [59.7–81.7]	<0.001	27.65	0.598	0.28	0.717	0.13
	PA		62.6 (6.0) [50.6–74.5]	472.4 (6.1) [60.3–84.5]						
Total BDI-II score	Non-PA	16.7 (2.0) [12.9–20.7]	11.2 (2.0) [7.3–15.2]	9.7 (2.0) [5.6–13.7]	<0.001	20.93	0.025	5.16	0.083	2.54
	PA	20.2 (2.4) [15.3–20.5]	13.2 (2.3) [13.7–17.6]	16.1 (2.3) [11.5–20.6]						
DEBQ restrained eating	Non-PA	28.0 (2.0) [24.2–31.9]	31.1 (2.0) [27.2–35.0]	30.6 (2.1) [26.5–34.6]	0.004	5.69	0.768	0.09	0.623	0.47
	PA	27.9 (2.5) [23.0–32.9]	32.9 (2.4) [28.2–37.7]	30.2 (2.5) [25.3–35.2]						
DEBQ emotional eating	Non-PA	36.1 (3.1) [30.0–42.1]	34.3 (3.1) [28.2–40.4]	30.3 (3.1) [24.1–36.5]	<0.001	10.66	0.330	0.96	0.410	0.90
	PA	35.5 (3.6) [28.3–42.6]	29.7 (3.5) [22.7–36.7]	27.6 (3.6) [20.5–34.6]						
DEBQ external eating	Non-PA	33.6 (1.4) [30.8–36.4]	29.3 (1.4) [26.5–32.1]	27.5 (1.4) [24.7–30.4]	<0.001	43.19	0.098	2.80	0.673	0.40
	PA	32.2 (1.7) [28.8–35.5]	26.7 (1.6) [23.5–29.8]	25.5 (1.6) [22.3–28.8]						

† Data are presented as estimated mean (SE) according to the mixed model analysis (p time- Time effect = analyze the changes over time in the two groups; p groups- Group effect = analyze between group differences; p time×group- Group time effect = analyze the interaction between the trend of change over time and the group effect). This model was adjusted for sociodemographic variables (sex, age, education level), surgery type and baseline BMI. Abbreviations: Body mass index (BMI), excess weight loss (EWL), 6 and 12 months post-operation (6 M, 12 M), Childhood Trauma Questionnaire (CTQ), Dutch Eating Behavior Questionnaire (DEBQ), emotional abuse (EA), physical neglect (PN), sexual abuse (SA), emotional neglect (EN), physical abuse (PA). ^1^ Data were available for 111, 101 and 73 participants at baseline, 6 M and 12 M, accordingly.

## Data Availability

De-identified data from this study can be made available on reasonable request (as allowable according to the host institution’s IRB) by emailing the corresponding author. Some of the materials used to conduct the REBORN study that constitute the larger bariatric cohort from which the current research project’s sample was derived are presented in a public archive at https://osf.io/qcsrt/, accessed on 21 April 2023.

## Supplementary Materials

The following supporting information can be downloaded at: https://www.mdpi.com/article/10.3390/nu15092046/s1, Figure S1: Evaluation of agreement level between self-reported measured weight (A) and height (B) at baseline across the study sample (*n* = 111) using a Bland–Altman plot; Table S1: Baseline sociodemographic characteristics of those who did and did not complete the Childhood Trauma Questionnaire (CTQ) at baseline within the REBORN cohort (*n* = 597); Table S2: Baseline characteristics of the study sample according to sex.

## Abbreviations

BDI-II: Beck Depression Inventory-II; BS, bariatric surgery; BMI, body mass index; CTQ, Childhood Trauma Questionnaire; DEBQ, Dutch Eating Behavior Questionnaire; EWL, excess weight loss.
