# Supplementary material for: Childhood Maltreatment in Patients Undergoing Bariatric Surgery: Implications for Weight Loss, Depression and Eating Behavior
Source: Nutrients. 2023 Apr 24;15(9):2046. doi: 10.3390/nu15092046 (PMC10181145; doi:10.3390/nu15092046)
Supplement: Supplementary file 1 [file nutrients-15-02046-s001.zip › nutrients-2344488-supplementary.pdf]

Table S1. Baseline sociodemographic characteristics of those who did and didn't complete the childhood trauma questionnaire [CTQ] at baseline within the REBORN cohort [n=597].

Parameter †	Completed CTQ [n=111]	Didn't complete CTQ [n=486]	p-value
Sex [n (%) females]	94 (84.7)	377 (77.6)	0.098
Age (years)	45.1 (11.7)	43.5 (11.2)	0.160
Race [n (% White)]	99 (89.2)	405 (83.3)	0.141
Education (years of studies)	14.0 (11.0-16.0)	13.0 (11.0-15.0)	0.186
Marital status			0.822
Single [n (%)]	30 (27.0)	144 (29.6)	
Common-law partner [n (%)]	28 (25.2)	106 (21.8)	
Married [n (%)]	44 (39.6)	187 (38.5)	
Divorced / separated [n (%)]	9 (8.1)	45 (9.3)	
Widowed [n (%)]	0 (0.0)	3 (0.6)	
Missing data	0 (0.0)	1 (0.2)	
Monthly income			0.126
Less than 23 000 $ [n (%)]	12 (10.9)	72 (14.8)	
23 001 $ - 37 000 $ [n (%)]	11 (10.0)	53 (10.9)	
37 001 $ - 57 000 $ [n (%)]	25 (22.7)	79 (16.3)	
57 001 $ - 84 000 $ [n (%)]	23 (20.9)	75 (15.4)	
More than 84 000 $ [n (%)]	29 (26.4)	116 (23.9)	
Don't know/ not answered [n (%)]	11 (9.9)	91 (18.8)	
Type 2 diabetes [n (%)]	25 (22.5)	116 (23.9)	0.323
Hypertension [n (%)]	45 (40.5)	185 (38.1)	0.893
High LDL cholesterol [n (%)]	29 (26.1)	107 (22.0)	0.306
Weight (kg)	129.0 (110.0-144.0)	131.5 (116.7-150.5)	0.083
Smoking Status [n, (%yes)]	11 (9.9)	58 (11.9)	0.538

†Values are expressed as the mean (SD) or median (25th–75th percentile) according to the variable.
Abbreviations: Body mass index (BMI), Childhood Trauma Questionnaire (CTQ), Low-density lipoprotein (LDL).

Figure S1. Evaluation of agreement level between self-reported measured weight [A] and height [B] at baseline across the study sample (n=111) using the Bland-Altman plot.

A. Bland-Altman plot of the measured and self-reported weight at baseline£

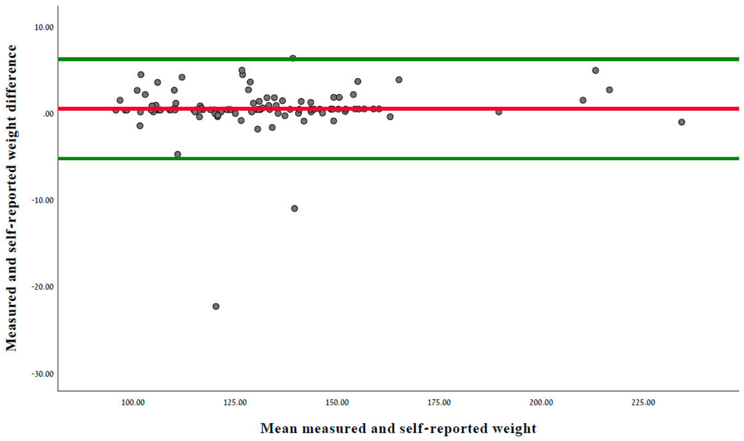

£As a first step, a one sample T test was performed (MD of measured and self-reported weight at baseline=0.352, SD=2.920, t=1.248, p=0.215), indicating that there was no statistically significant difference between measured and self-reported weight at baseline. As a second step a Bland-Altman plot was conducted, as shown above, with a mean difference= 0.35 (red line), Upper 95% CI= 6.08 (upper green line), Lower 95% CI= -5.37 (lower green line)], indicating a reasonably high accuracy between self-reported and measured weight. Finally, a linear regression model indicated no statistically significance [B=0.009, t=0.760, p=0.449], suggesting that there was no systematic bias across the weight spectrum.

B. Bland-Altman plot of the measured and self-reported height at baseline£

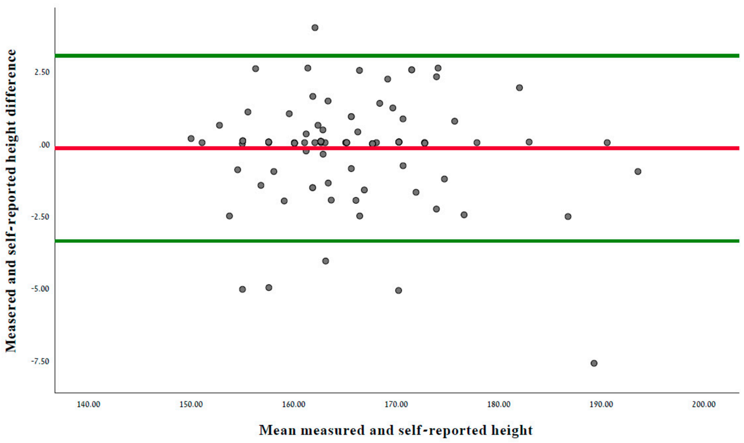

£As a first step, a one sample T test was performed (MD of measured and self-reported height at baseline=-0.196, SD=1.635, t=-1.265, p=0.208), indicating that there was no statistically significant difference between measured and self-reported height at baseline. As a second step a Bland-Altman plot was conducted, as shown above, with a mean difference= -0.19 (red line), Upper 95% CI= 3.01 (upper green line), Lower 95% CI= -3.40 (lower green line)], indicating a reasonably high accuracy between self-reported and measured height. Finally, a linear regression model indicated no statistically significance [B=-0.180, t=-0.944, p=0.347], suggesting that there was no systematic bias across the height spectrum.

Table S2. Baseline characteristics of the study sample according to sex.

Parameter †	Female [n=94, 84.7%]	Male [n=17, 15.3%]	p-value
Sociodemographic and medical background (n=111)			
Age (years)	44.4 (11.9)	49.3 (9.7)	0.128
Race [n (% White)]	83 (88.3)	16 (94.1)	0.477
Education (years of studies)	14.0 (11.0-16.0)	13.0 (11.0-14.5)	0.537
Marital status<br>Single [n (%)]<br>Common-law partner [n (%)]<br>Married [n (%)]<br>Divorced / separated [n (%)]<br>Widowed [n (%)]	 26 (27.7)<br>25 (26.6)<br>37 (39.4)<br>6 (6.4)<br>0 (0.0)	 4 (23.5)<br>3 (17.6)<br>7 (41.2)<br>3 (17.6)<br>0 (0.0)	0.421
Monthly income<br>Less than 23 000 $ [n (%)]<br>23 001 $ - 37 000 $ [n (%)]<br>37 001 $ - 57 000 $ [n (%)]<br>57 001 $ - 84 000 $ [n (%)]<br>More than 84 000 $ [n (%)]<br>Don't know/ not answered [n (%)]	 11 (11.8)<br>8 (8.6)<br>22 (23.7)<br>20 (21.5)<br>22 (23.7)<br>11 (11.7)	 1 (5.9)<br>3 (17.6)<br>3 (17.6)<br>3 (17.6)<br>7 (41.2)<br>0 (0.0)	0.485
Type 2 diabetes [n (%)]	16 (17.0)	9 (52.9)	**0.001**
Hypertension [n (%)]	35 (37.2)	10 (58.8)	0.238
High LDL cholesterol [n (%)]	19 (20.2)	10 (58.8)	**0.004**
Currently seeing psychologist/ psychiatrist [n (%)]	26 (27.7)	3 (17.6)	0.387
Weight (kg)	124.3 (109.1-137.9)	154.2 (146.3-183)	**<0.001**
BMI (kg/m^2)	46.2 (42.3-51.3)	52.5 (45.6-55.1)	**0.023**
Smoking Status [n, (%yes)]	9 (9.6)	2 (11.8)	0.781
History of childhood maltreatment based on CTQ scores (n=111)			
Total CTQ Score	38.0 (31.0-55.5)	40.0 (26.0-51.0)	0.392
Emotional Abuse [scale Score]	8.0 (6.0-12.0)	8.0 (5.0-12.0)	0.611
Physical Abuse [scale Score]	5.0 (5.0-8.0)	5.0 (5.0-7.0)	0.818
Emotional Neglect [scale Score]	9.5 (7-15)	12.0 (5-15)	0.876
Physical Neglect [scale Score]	7.0 (5.0-9.0)	7.0 (5.0-9.0)	0.913
Sexual Abuse [scale Score]	5.0 (5.0-9.3)	5.0 (5.0-5.0)	**0.014**
Eating behavior based on DEBQ scores (n=79)			
DEBQ Restrained eating scale	29.0 (24.2-31.8)	28.0 (24.0-30.0)	0.470
DEBQ Emotional eating scale	40.8 (12.4)	28.6 (11.7)	**0.002**
DEBQ External eating scale	32.2 (5.2)	30.2 (6.1)	0.206
Depressive symptoms based on BDI-II scores (n=85)			
BDI-II total score	15.0 (8.0-21.0)	13.0 (10.2-17.5)	0.975
Symptomology classifications<br>Minimal depression [n (%)]<br>Mild depression [n (%)]<br>Moderate depression [n (%)]<br>Severe depression [n (%)]	 33 (45.2)<br>18 (24.7)<br>14 (19.2)<br>8 (11.0)	 6 (50.0)<br>3 (25.0)<br>2 (16.7)<br>1 (8.3)	0.985

†Values are expressed as the mean (SD) or median (25th–75th percentile) according to the variable.
Abbreviations: BDI-II, Beck Depression Inventory-II; BMI, Body mass index (BMI); CTQ; Childhood Trauma Questionnaire; DEBQ, Dutch Eating Behavior Questionnaire; LDL, Low-density lipoprotein.
